# The Triangular Interaction Between Dietary Polyphenols, Gut Microbiota and Type 2 Diabetes

**DOI:** 10.3390/ijms27114782

**Published:** 2026-05-26

**Authors:** Emily Bayliss, Landri Shope, Seth Woodfin, Gretchka Mair, Matthew H. Becker, William Moore

**Affiliations:** 1 School of Health Sciences, Department of Biology and Chemistry, Liberty University, Lynchburg, VA 24515, USA; egbayliss@liberty.edu (E.B.); lcshope@liberty.edu (L.S.); gmmair@liberty.edu (G.M.); mbecker5@liberty.edu (M.H.B.); 2 Department of Biomedical Sciences, West Virginia School of Osteopathic Medicine, Lewisburg, WV 24901, USA; swoodfin@osteo.wvsom.edu

**Keywords:** type 2 diabetes mellitus (T2D), gut microbiome, polyphenols, bioavailability and metabolism, insulin resistance, short-chain fatty acids (SCFAs), oxidative stress, inflammation, host-microbiome interactions, precision nutrition

## Abstract

Type 2 diabetes (T2D) is a growing global health concern characterized by peripheral insulin resistance and impaired insulin secretion from pancreatic β-cells. Emerging evidence suggests that the gut microbiome, specifically gut dysbiosis, defined as an imbalance in the gut microbial composition and function, is a critical modulator of the pathophysiology of T2D. Dietary polyphenols, a diverse group of bioactive compounds that are abundant in plant-based foods, have gained increasing attention for their potential to attenuate metabolic disorders through their antioxidant and anti-inflammatory properties. These compounds work through the modulation of gut microbial composition and activity. This process effectively ameliorates dysbiosis. However, the diabetic state itself may influence polyphenol metabolism, absorption, and bioavailability, potentially limiting their therapeutic efficacy. This review examines the complex interrelationships between T2D, dietary polyphenols, and the gut microbiota and proposes a dynamic triangular interaction between these factors that might inform novel strategies for the prevention and management of metabolic disease.

## 1. Introduction

More than two thousand years ago, the Ancient Greek physician Hippocrates asserted that “All disease begins in the gut.” This statement has proven to be remarkably prescient, as evidenced by a growing body of scientific literature examining the relationship between the gut microbiome and the pathogenesis of metabolic diseases, including type 2 diabetes mellitus (T2D) [1]. T2D is a chronic metabolic disease that ranks among the top ten causes of death globally, according to the World Health Organization [2]. In 2022, T2D and its associated complications accounted for an estimated $413 billion in total medical costs, lost productivity, and wages in the United States, making it the most costly chronic disease nationally [3,4]. Since 2000, mortality attributed to T2D in the U.S. has increased by approximately 95% [2]. Representing a substantial public health burden, more than 90% of diabetes cases worldwide are classified as type 2, according to the International Diabetes Federation [5].

T2D is characterized by chronically elevated fasting plasma glucose concentrations (hyperglycemia), systemic insulin resistance, and widespread metabolic dysregulation [6]. Disease pathogenesis arises from a combination of impaired insulin secretion by pancreatic β-cells and diminished responsiveness of insulin-sensitive tissues to circulating insulin [7]. Insulin resistance promotes hyperglycemia through reduced glucose uptake in skeletal muscle, liver, and adipose tissue, as well as increased hepatic glucose production [8]. Individuals with T2D commonly exhibit increased adiposity, particularly distributed in the abdominal region [7], and may experience classic symptoms such as polyuria, polydipsia, and polyphagia [9].

Multiple non-modifiable risk factors contribute to the development of T2D, including aging, genetic predisposition, epigenetic influences, and ethnicity [10]. Lifestyle-related risk factors include chronic overnutrition, sedentary lifestyle, and smoking [7]. T2D is a progressive condition that substantially increases the risk of macrovascular complications such as coronary artery disease, peripheral artery disease, and stroke, as well as microvascular complications, including retinopathy, nephropathy, and neuropathy [11]. Although many cases of T2D are largely preventable through lifestyle modification, identifying effective strategies for disease prevention and long-term management remains a critical public health priority [12].

Conventional management of T2D relies on a combination of lifestyle and medical interventions, including structured exercise programs, dietary modifications, pharmacotherapy, bariatric surgery, and multifactorial treatment approaches, all aimed at achieving and maintaining glycemic control [13]. Increasing evidence indicates that the gut microbiome plays a central role in host metabolic regulation and energy homeostasis [14]. Notably, gut dysbiosis, defined as alterations in the composition and functional capacity of the intestinal microbiota, has been closely linked to the development and progression of T2D [15]. As a regulator of metabolic homeostasis, the gut microbiome has emerged as a promising therapeutic target for both the treatment and prevention of T2D [13].

From a clinical perspective, the management of T2D is guided by well-established evidence-based recommendations that prioritize glycemic control and reduction in cardiovascular risk. Current guidelines from the American Diabetes Association (ADA) and other international organizations emphasize the importance of early diagnosis through routine screening of high-risk individuals, followed by a stepwise treatment approach beginning with lifestyle modifications and metformin therapy [16,17]. If glycemic targets are not achieved, additional pharmacologic agents, including glucagon-like peptide-1 receptor agonists, sodium-glucose cotransporter-2 inhibitors, and other antihyperglycemic medications, are introduced based on patient characteristics, such as comorbidities, cardiovascular risk, and renal function [18]. Despite these advances, many individuals continue to experience progressive metabolic dysfunction and ultimately require multiple pharmacologic agents to achieve adequate glycemic control. Consequently, there is increasing interest in identifying adjunctive strategies that target underlying metabolic and inflammatory pathways implicated in disease progression [19,20]. In this context, emerging research linking the gut microbiota, metabolic regulation, and dietary bioactive compounds offers clinically relevant insight into novel approaches that may complement existing therapeutic frameworks [21].

Dietary polyphenols have attracted significant interest as potential modulators of metabolic health. These compounds are secondary plant metabolites found abundantly in foods such as coffee, tea, fruits, vegetables, cereals, and wine [20]. Structurally, polyphenols contain one or more aromatic rings with single or multiple hydroxyl groups and are widely recognized for their bioactive properties. Many polyphenols exhibit antioxidant activity through their ability to neutralize reactive oxygen species (ROS) [22]. Their reported health-promoting effects include anti-inflammatory, antioxidant, neuroprotective, anti-adipogenic, and antibacterial actions [22].

The biological effects of dietary polyphenols are influenced by their bioavailability, defined as the proportion of an ingested compound that reaches the systemic circulation and target tissues where biological activity can be exerted [23]. Notably, many polyphenols undergo extensive metabolism by the microbiota, which converts them into smaller, more bioactive, and readily absorbable metabolites [20]. In addition, polyphenols can directly modulate gut microbial composition by promoting the growth of beneficial bacterial taxa while suppressing potentially pathogenic microorganisms [24]. For example, diets that are rich in polyphenols have been shown to influence the relative abundance of Bacillota and Bacteroidota within the gut microbiome [24]. Because a substantial proportion of dietary polyphenols escapes absorption in the small intestine, these compounds accumulate in the large intestine, where they serve as substrates for microbial metabolism.

The ability of dietary polyphenols to influence gut microbial composition and function suggests a promising therapeutic avenue for the management of T2D. This review examines current evidence linking gut microbiota, dietary polyphenols, and T2D and proposes a dynamic triangular interaction among these factors. While substantial evidence supports the role of polyphenols in modulating gut microbiota and improving metabolic outcomes, disentangling the complex relationships among gut dysbiosis, insulin resistance, and polyphenol metabolism remains challenging. Dietary patterns, interindividual variability, genetic predisposition, medication use, and other lifestyle factors can all contribute to disease development and progression and must be considered when interpreting existing findings [25].

Despite substantial advances in pharmacotherapy and lifestyle interventions, current treatments often fall short of adequately addressing underlying gut dysbiosis, chronic low-grade inflammation, and oxidative stress, highlighting the need for complementary strategies such as dietary polyphenol interventions that target the gut microbiota-metabolism axis [26].

We hypothesize that this triangular interaction is bidirectional: dietary polyphenols reshape gut microbial composition and function to exert beneficial metabolic effects in T2D, while the diabetic state and associated dysbiosis reciprocally impair polyphenol bioavailability and metabolism. This framework provides a basis for developing targeted polyphenol-based and microbiome-modulating strategies in the prevention and management of T2D [27]. 

## 2. Current T2D Treatments

Effective glycemic control is foundational to the management of T2D. While lifestyle interventions and pharmacologic therapies are critical components of treatment, the progressive nature of the disease means that many individuals will eventually need an antihyperglycemic medication to maintain metabolic control [28]. Metformin, the recommended first-line agent for T2D management, works by suppressing hepatic gluconeogenesis [29]. Common adverse effects of metformin include gastrointestinal symptoms such as vomiting, nausea, and abdominal discomfort, which can often be minimized by gradual dose escalation [30]. It has also been associated with an increase in the risk of vitamin B12 malabsorption and, in rare cases, lactic acidosis [25].

Sulfonylureas promote insulin secretion by binding to ATP-sensitive potassium channels on pancreatic β-cell receptors, leading to membrane depolarization, calcium influx, and subsequent exocytosis of insulin-containing secretory granules [31]. While effective in terms of lowering blood glucose, sulfonylureas are associated with an increased risk of hypoglycemia, particularly due to their prolonged duration of action. Additionally, limited selectivity and off-target effects, including binding to potassium channels in cardiomyocytes, could increase risk of adverse cardiovascular events [32]. While these conventional antihyperglycemic treatments are effective and widely accessible, their potential adverse effects and diminishing efficacy over time underscore the need for safer and more targeted approaches [33].

In contemporary clinical practice, pharmacologic management of T2D increasingly emphasizes the treatment of patient-specific comorbidities rather than glucose lowering alone. Current clinical guidelines recommend selecting antihyperglycemic therapies based on the presence of conditions such as atherosclerotic cardiovascular disease, heart failure, or chronic kidney disease, as several drug classes have conferred cardiovascular and renal protective effects that extend beyond glycemic control [34]. For example, glucagon-like peptide-1 (GLP-1) receptor agonists have been shown to reduce the incidence of major adverse cardiovascular events, while sodium-glucose cotransporter 2 (SGLT2) inhibitors significantly lower the risk of hospitalization for heart failure and slow the progression of diabetic kidney disease [35]. As a result, treatment selection increasingly incorporates broader cardiometabolic risk profiles when developing individualized therapeutic strategies.

Despite notable advances in pharmacotherapy, the long-term management of T2D remains challenging. As disease severity progresses, many patients require increasingly complex regimens involving multiple antihyperglycemic agents and, in some cases, insulin therapy, to maintain metabolic control [36]. This therapeutic complexity can lead to reduced medication adherence, increase treatment cost, and elevated risk of adverse effects. Additionally, individuals with T2D often require concurrent pharmacologic management of comorbid conditions such as hypertension and dyslipidemia, further increasing the risk of polypharmacy [37]. Together, these clinical challenges highlight the need for safe, complementary strategies that target underlying pathophysiological mechanisms, particularly gut dysbiosis, chronic inflammation, and oxidative stress, while minimizing additional pharmacologic complexity [36,37].

## 3. Gut Microbiota

### 3.1. Taxonomy and Function

The gut microbiota (GM) refers to the diverse microorganisms that inhabit the gastrointestinal tract and collectively contribute to host homeostasis and metabolic regulation. In contrast, the gut microbiome refers to the aggregate of microbial genomes, their associated metabolites, environmental conditions, and structural elements within this ecosystem [38]. Although the number of microorganisms in the GI tract has been estimated to exceed 10^14^, a recently revised estimate of microbial abundance suggests that the human-to-bacterial cell ratio is approximately 1:1 [39]. In addition to bacteria, the gut microbiota includes fungi, viruses, protozoa, and archaea, each contributing to the functional capacity of the microbial community [40].

The gut microbiota performs a variety of essential physiological functions, such as regulating host immunity, protecting against pathogens, maintaining the integrity of the intestinal epithelium, harvesting energy, synthesizing vitamins, and participating in carbohydrate metabolism [8,38,39].

In healthy individuals, the gut microbiota is dominated by six major bacterial phyla: Bacillota, Bacteroidota, Actinomycetota, Pseudomonadota, Fusobacteriota, and Verrucomicrobiota. Among these, Bacillota and Bacteroidota play a dominant role in shaping an individual’s unique gut microbial profile [8].

### 3.2. Gut Dysbiosis and Type 2 Diabetes

Gut dysbiosis is increasingly recognized as a contributing factor to the onset and progression of T2D. Metagenomic sequencing analyses have shown that reduced microbial diversity is associated with conditions such as obesity and inflammatory bowel disease [41,42]. The Bacillota/Bacteroidota (F/B) ratio has been proposed as a marker of obesity or gut dysbiosis, but findings are inconsistent across studies [43]. Alterations in this ratio have been linked to heightened inflammation and oxidative stress, both of which are central features of early disruptions in gut homeostasis in diabetes [1].

Imbalances in the composition and function of the gut microbiota have been implicated in the pathogenesis of T2D, obesity, and cardiovascular disease [8]. Clinical studies demonstrate that the human gut microbiome influences insulin sensitivity and circulating metabolite profiles. Individuals with insulin resistance exhibit fasting serum metabolomes enriched in branched-chain amino acids (BCAAs), which correlate with microbial communities demonstrating increased potential for BCAA biosynthesis [8]. Increased serum BCAA levels have likewise been associated with the development of insulin resistance [44,45].

Nonetheless, alterations in gut microbiota composition have also been associated with increased intestinal permeability and the translocation of microbial components, such as lipopolysaccharide (LPS), into systemic circulation. This process can promote chronic low-grade inflammation and activate intracellular inflammatory signaling pathways implicated in the development of insulin resistance. Two major pathways that are activated during these responses are the stress-activated c-Jun N-terminal kinase (JNK) and the nuclear factor κB (NF-κB) signaling cascades. Activation of these pathways induces the transcription of pro-inflammatory genes and stimulates the production of cytokines such as interleukin-1 (IL-1), interleukin-6 (IL-6), tumor necrosis factor-α (TNF-α), and various chemokines [46]. Deviations in microbial homeostasis can therefore promote widespread inflammatory signaling, compromise intestinal barrier integrity, and impair immune tolerance [8]. Interestingly, the gastrointestinal tract is the only site in the human body where the immune system is continuously activated by environmental stimuli [47].

Gut dysbiosis can also induce the production of ROS via the stimulation of formyl peptide receptors (FPRs). ROS are highly electrophilic molecules that include both radical species such as superoxide (O_2_^−^), and non-radical species such as hydrogen peroxide (H_2_O_2_) [48]. While high concentrations of ROS are toxic to microbes and are used by phagocytes to eliminate pathogens, epithelial ROS production may serve a conserved role in maintaining bacterial-mediated gut homeostasis. These molecules can function as intra- and intercellular messengers to regulate an immune response [49].

Substantial evidence suggests that insulin resistance can be modulated through fecal microbiota transplantation (FMT), emphasizing the therapeutic potential of microbe-targeted strategies for T2D [50,51]. FMT involves transferring stool from a healthy donor in order to normalize the gut microbial composition of the recipient. Notably, the same approach can also transfer insulin-resistant phenotypes, further demonstrating the strong link between microbial communities and human metabolic function [47].

Clinically, the growing body of evidence linking gut dysbiosis to metabolic disease has generated interest in microbiome-based diagnostic and therapeutic strategies. However, the clinical application of these findings remains limited. While FMT has demonstrated efficacy in the treatment of recurrent *Clostridioides difficile* infection, its use in metabolic disorders, including T2D, remains largely investigational and is not currently recommended in routine clinical practice [52,53]. Similarly, although advances in metagenomic sequencing technologies have enhanced the characterization of the gut microbiome, microbiome profiling has not yet been incorporated into standard diabetes screening or management guidelines [54]. Nevertheless, continued research into host-microbiome interactions may eventually facilitate the development of microbiome-based biomarkers capable of identifying individuals at increased metabolic risk or predicting their response to specific therapeutic interventions. As our understanding of these complex microbial ecosystems expands, microbiome-targeted interventions may emerge as complementary strategies in T2D prevention and management.

### 3.3. SCFAs and Metabolic Health

The gut microbiota metabolizes dietary polysaccharides to produce monosaccharides and short-chain fatty acids (SCFAs), which exert local effects in the colon and systemic effects after being absorbed into the circulation [8]. Dysbiosis-associated alterations in microbial metabolites, such as SCFAs, bile acids, and endotoxins such as LPS, play an important role in the pathogenesis of T2D. These metabolites influence pathways involved in pancreatic β-cell function, insulin sensitivity, and systemic inflammation [55]. Reducing SCFA production may compromise gut barrier integrity, which allows bacteria and bacterial metabolites to translocate into circulation and serve as an early indicator of disrupted metabolic homeostasis, including diminished insulin activity and increased adiposity [1].

SCFAs, produced through microbial fermentation of dietary carbohydrates, have been proposed to influence glucose homeostasis and insulin sensitivity [56]. SCFAs may improve glycemic control by increasing the secretion of GLP-1, thereby improving glucose tolerance and insulin responsiveness. They also contribute to gut barrier integrity through the induction of mucin synthesis and tight junction assembly. In addition, SCFAs promote satiety by promoting secretion of peptide YY and leptin and play key roles in host immune regulation [8].

One hallmark of T2D-associated dysbiosis is reduced production of butyrate, which is a SCFA with important metabolic and anti-inflammatory functions. Dietary fiber, which serves as the primary substrate for microbial SCFA production, is already emphasized in clinical nutrition guidelines for individuals with diabetes due to its beneficial effects on glycemic regulation and cardiovascular risk [57]. Butyrate supports pancreatic β-cell function in the postprandial state [8], protects β-cells from cytokine-induced dysfunction, and has been shown to upregulate genes associated with secretion and transport [58]. Additionally, butyrate has also been shown to improve metabolic health and reduce inflammation in insulin-sensitive tissues, further supporting its potential anti-diabetic effects [59].

## 4. Dietary Polyphenols

### 4.1. Polyphenol Classification

Dietary polyphenols are classified into five primary categories based on their chemical structure: flavonoids, phenolic acids, stilbenes, lignans, and tannins [60]. These compounds are broadly divided into flavonoid-type phenolics, which are characterized by a core structure consisting of two benzene rings (A and B) connected by a heterocyclic pyrone C ring, and non-flavonoid phenolics, which encompass the remaining four structurally diverse categories [20]. The major classes of polyphenols are summarized in Table 1, including their chemical features, food sources, microbial metabolism, bioavailability challenges in T2D, and beneficial mechanisms [61].

#### 4.1.1. Flavonoids

Flavonoids are further subclassified according to the attachment position of the B ring to the C ring, as well as the degree of oxidation and saturation of the C ring. The major subclasses include flavanols, flavones, flavanones, isoflavones, anthocyanins, and chalcones [62]. This class of low-molecular-weight polyphenols shares a common C6-C3-C6 structural skeleton, which consists of a phenyl ring linked to a benzo-γ-pyrone structure [60,63].

Flavonoids have been demonstrated to regulate hepatic enzyme activities, glucose metabolism, and lipid profiles, thus modulating disease progression and complications associated with T2D [64]. Their anti-diabetic effects are primarily exerted by activating AMP-activated protein kinase (AMPK), promoting GLUT4 translocation and glucose uptake, and suppressing pro-inflammatory NF-κB signaling and cytokine production [65]. 

#### 4.1.2. Phenolic Acids

Phenolic acids are characterized by a single phenolic ring and an aromatic carboxylic acid moiety and frequently exist in planta as esters, amides, or glycosides [60]. Hydrolysis by host or microbial esterases is required to liberate phenolic acids from their bound forms, enabling the unbound compound to be absorbed into systemic circulation [20].

Hydrocinnamic acids, a subgroup of phenolic acids that includes chlorogenic acid, ferulic acid, and caffeic acid, have been identified as key microbial metabolites associated with improvements in lipid metabolism and reductions in hepatic steatosis through activation of the AMPK signaling pathway [66]. These findings support the potential role of phenolic acids in mitigating obesity-related metabolic dysfunction.

#### 4.1.3. Stilbenes

Stilbenes are structurally characterized by a 1,2-diphenylethylene backbone containing two phenolic rings connected by a methylene bridge and are derived from the phenylpropanoid pathway [60]. This class is most commonly represented by resveratrol, which is typically ingested in vivo as its glycosylated precursor, trans-piceid, and subsequently metabolized by the gut microbiota. Resveratrol has received considerable attention due to its chemoprotective, neuroprotective, and anti-inflammatory properties [67]. Resveratrol and other stilbenes exert beneficial effects largely via SIRT1 activation, enhancement of insulin sensitivity, and reduction in oxidative stress and inflammation [68].

#### 4.1.4. Lignans

Lignans are widely classified as phytoestrogens due to their structural similarity to endogenous steroid hormones [69]. In addition to their established role in reducing the risk of cancer, lignans have been identified as compounds with antidiabetic potential. They may contribute to the management of T2D by lowering blood glucose concentrations and enhancing insulin sensitivity [70].

When ingested, lignans undergo extensive microbial metabolism, primarily demethylation and dihydroxylation by specific gut bacteria (i.e., *Eggerthella* and *Clostridium* spp.) [69], converting them into more bioactive and absorbable enterolignans. For example, matairesinol and secoisolariciresinol are converted by intestinal bacteria into enterolactone and enterodiol, respectively [20]. Following microbial conversion to enterolignans, these compounds act as phytoestrogens that enhance insulin sensitivity and modulate glucose and lipid metabolism [71]. 

#### 4.1.5. Tannins

Tannins are classified into two major groups: hydrolysable tannins and condensed tannins. Hydrolysable tannins are polyesters formed from gallic or ellagic acid esterified to a carbohydrate core, typically glucose, and can be degraded by specific enzymes as well as acidic or basic conditions [60]. Condensed tannins, which include proanthocyanidins, consist of high molecular weight polymeric flavan-3-ols and are distinguished by their strong protein-binding capacity [72]. Tannins contribute to metabolic benefits through antioxidant activity, modulation of gut microbiota composition, and increased production of SCFAs (especially butyrate), thereby improving insulin sensitivity and reducing inflammation [73]. 

Because polyphenol biological activity is closely tied to chemical structure, understanding structural diversity is critical for evaluating antioxidant capacity and metabolic effects relevant to T2D. These relationships are explored further in subsequent sections. Structural classification is important because the degree of polymerization and type of tannin strongly influence their bioavailability, microbial metabolism, and ability to exert antioxidant and prebiotic effects in the gut [74]. 

In addition to their direct biochemical properties, dietary polyphenols exert important effects through extensive biotransformation by the gut microbiota and modulation of microbial composition, as summarized in Table 1 and described in the following subsections [75].

### 4.2. Polyphenol Properties

Polyphenols exhibit substantial immunomodulatory activity, particularly in reducing intestinal inflammation linked to gut dysbiosis. For example, chlorogenic acid exerts anti-inflammatory effects by modulating the macrophage NOD-, LRR-, and pyrin domain-containing protein 3 (Nlrp3) inflammasome pathway and preventing pro-inflammatory M1 macrophage polarization by inhibiting pyruvate kinase M2 (PKM2)-dependent glycolysis. Similarly, urolithin A, a microbial metabolite of ellagic acid, enhances gut barrier integrity via an interleukin-22 (IL-22)-dependent mechanism [76]. Collectively, these findings underscore the ability of polyphenols to regulate immune and metabolic pathways relevant to T2D pathogenesis [77].

### 4.3. Polyphenol Biotransformation and Absorption

Dietary polyphenols undergo extensive biotransformation within the gastrointestinal tract, a process that influences their bioavailability, bioactivity, solubility, and stability. The reciprocal relationship between microbial biotransformation of polyphenols and polyphenol-induced modulation of gut microbiota composition has been associated with improved health. Notably, the most abundant dietary polyphenols are not necessarily the most bioavailable. There are a variety of host-related factors that affect xenobiotic metabolism, including intestinal enzymatic activity, transit time, microbial composition, disease states, and physiological conditions [23].

Most dietary polyphenols are not readily absorbed in their native form because they are typically present as glycosides or other large, complex structures. The gut microbiota plays a critical role in breaking these compounds down through deglycosylation and other reactions, generating smaller, more absorbable metabolites such as aglycones and phenolic acids. These low-molecular-weight metabolites primarily cross the colonic epithelium by passive transcellular diffusion due to their increased lipophilicity, although some hydrophilic metabolites may also utilize paracellular pathways. By converting poorly absorbed parent compounds into more absorbable forms, microbial metabolism substantially enhances systemic exposure and contributes to the antioxidant, anti-inflammatory, and metabolic benefits associated with dietary polyphenols [78].

Only 5–10% of dietary polyphenols, primarily monomeric and dimeric forms, are directly absorbed in the small intestine. These compounds typically undergo deconjugation reactions, such as deglycosylation, followed by Phase I reactions (oxidation, reduction, hydrolysis) and Phase II conjugation reactions, including methylation, sulfation, and glucuronidation [79].

Initial metabolism occurs in enterocytes and is followed by hepatic processing, resulting in water-soluble metabolites that are either released into systemic circulation to or excreted. The remaining 90–95% of dietary polyphenols that escape small intestinal absorption reach the colon, where microbial enzymes cleave glycosidic linkages and degrade heterocyclic structures [79].

Three main structural properties influence intestinal absorption of polyphenols: molecular weight, degree of glycosylation, and esterification [80]. High-molecular-weight polyphenols, including oxidized polymeric polyphenols, are poorly absorbed, while lower-molecular-weight compounds, such as phenolic acids, exhibit greater bioavailability [79,80]. Colonic microbiota are responsible for the catabolism of these unabsorbed polyphenols into lower-molecular-weight phenolic metabolites, which may be responsible for many of the observed health benefits associated with polyphenol-rich diets; For example, ellagitannins can be broken down by gut bacteria into urolithins [79]. Notably, proanthocyanins, with a degree of polymerization greater than four (DP > 4), are considered essentially unabsorbed due to steric limitations [81].

All polyphenols function as reducing agents and therefore contribute to cellular protection cells against oxidative damage. Their redox potential is dependent on structure, which compounds containing adjacent (vicinal) hydroxyl groups exhibiting enhanced free radical scavenging capacity relative to those with fewer hydroxyl substitutions. However, differences in antioxidant capacity are often outweighed by variation in intestinal absorption efficiency.

In plants, polyphenols, particularly flavonoids, commonly often occur as water-soluble O- or C-glycosides [22,82]. In these conjugates, the sugar moiety is referred to as the glycone, while the polyphenol is known as the aglycone [23]. Deglycosylation by intestinal enzymes or gut microbial β-glucosidases is typically required for absorption and subsequent Phase I hepatic metabolism [82].

Resveratrol is commonly ingested in its glycosylated form, polydatin (piceid). Due to its larger molecular size, polydatin shows lower bioavailability than the aglycone and requires enzymatic hydrolysis by intestinal or microbial β-glucosidases to generate the absorbable form [83,84]. This example highlights how the glycosylation state of polyphenols significantly influences their intestinal absorption and subsequent bioactivity. 

Members of the gut microbiota within the Lachnospiraceae, Lactobacillaceae, and Bifidobacteriaceae families are known to hydrolyze flavonoid O-glycosides, while the cleavage of C-glycosides has been attributed primarily to Streptococcaceae, Enterococcaceae, and Lachnospiraceae species [70]. Deglycosylation in the host is facilitated by lactase-phlorizin hydrolase and cytosolic β-glucosidase, which are expressed in the intestinal epithelium. While both host and gut microbial enzymes contribute to polyphenol deglycosylation, the necessity of enzymatic cleavage is essential for absorption is still under debate [70].

Anthocyanins represent an exception as they can be absorbed in glycosylated form due to the intrinsic instability of their aglycone structures, which necessitates alternative metabolic pathways [23]. Esterification can also influence the intestinal absorption of polyphenols. Because there are no human esterases that can cleave these linkages, colonic microbiota serve as the primary site of metabolism for these compounds [85].

Before entering systemic circulation, polyphenols undergo additional conjugation reactions in the intestine and liver, including sulfation, glucuronidation, and methylation. These modifications increase solubility and molecular weight, facilitating biliary and urinary excretion and representing classic detoxification pathways in xenobiotic metabolism [23].

While many studies have investigated the biological effects of polyphenols in their native forms, accumulating evidence suggests that microbial metabolites may contribute equally, or even predominantly, to their physiological effects. Consequently, the biotransformation of dietary polyphenols by both the host and gut microbiota is an important determinant of their health-promoting potential [86].

### 4.4. Modulation of Gut Microbiota by Dietary Polyphenols

Polyphenols can beneficially modulate gut microbiota composition and function. A recent study demonstrated that extract from *Glycyrrhiza glabra* (licorice root), which is rich in flavanols, isoflavones, and chalcones, improved insulin resistance, serum lipid profiles, and endotoxemia-related inflammation in type 2 diabetic mice [87]. The extract promoted the growth of health-associated bacterial genera including *Alloprevotella*, *Bacteroides*, and *Akkermansia*, while reducing the abundance of Lachnospiraceae NK4A136 group, a taxon that is positively correlated with T2D. Additionally, the ratio of Bacillota: Bacteroidota decreased, and was accompanied by significant reductions in colonic IL-6, IL-12, and TNF-α expression [87].

Similar effects were observed in high-fat, sucrose-fed rats, in which quercetin attenuated microbiota dysbiosis and resveratrol modulated mRNA expression of tight-junction proteins and inflammation-associated genes, including toll-like receptor 4 (TLR4), lipopolysaccharide binding protein (LBP), and interleukin 18 (IL-18). Quercetin supplementation decreased Bacillota abundance by 34.2% (not statistically significant) and reduced the Bacillota: Bacteroidota ratio by 80.5%. Both compounds improved insulin sensitivity and decreased insulin levels, which supports their potential roles as antidiabetic agents [88].

Microbial metabolites of polyphenols also exhibit anti-inflammatory effects. In endothelial HMEC-1 cells exposed to hyperglycemia, these metabolites reduced IL-6, indicating mitigation of hyperglycemia-induced endothelial inflammation [89]. In vivo, polyphenol supplementation has been shown to reduce lipid deposition and improve insulin sensitivity in high-fat diet-fed rats and Ningxiang pigs, partly through the enrichment of *Akkermansia muciniphila* and increased relative abundance of Bacteroidota [90].

Although the data support the beneficial role of polyphenols in improving glucose homeostasis through microbiota modulation, the underlying mechanisms are not fully elucidated. The complexity and bidirectionality of polyphenol-microbiome interactions present both challenges and opportunities for future translational research and therapeutic development [91]. The following sections build upon these foundational properties by examining clinical evidence in T2D (Section 5) and the influence of the diabetic state on polyphenol pharmacokinetics (Section 6). 

## 5. Polyphenols and T2D

While Section 4 focused on the classification, structural features, biotransformation, and general mechanisms of dietary polyphenols, this section examines the specific evidence linking polyphenol consumption to improved outcomes in T2D from observational and interventional studies. Conventional treatments for T2D are effective but often accompanied by adverse effects, highlighting the need for complementary therapies that can support metabolic health while minimizing treatment burden. Polyphenols, which are abundant antioxidants found in numerous plant-based foods, have been proposed as promising antidiabetic agents [92].

Many polyphenol-rich foods, including berries, tea, cocoa, coffee, and olive oil, are key components of dietary patterns that have been associated with improved cardiometabolic health in both observational and interventional studies [93]. Consequently, clinicians often encourage patients with T2D to adopt plant-forward dietary approaches, including the Mediterranean diet, which has demonstrated benefits for glycemic regulation and cardiovascular risk reduction [93]. While these benefits cannot be attributed exclusively to polyphenols, growing evidence suggests that these bioactive compounds may contribute to the metabolic improvements observed [94].

Despite these encouraging findings, the clinical application of polyphenol-based interventions remains limited. Many studies examining the effects of polyphenols on glucose metabolism have been conducted in animal models or small human cohorts, and standardized dosing strategies have not yet been established. In addition, the bioavailability and metabolic impact of dietary polyphenols can vary widely depending on interindividual differences in gut microbiota composition, dietary habits, and host metabolism [91]. As a result, polyphenol supplementation cannot currently be recommended as a stand-alone therapy for T2D. Nevertheless, continued investigation into the interplay between dietary polyphenols and gut microbiota may inform future nutritional or pharmacologic strategies aimed at improving metabolic health [91].

While no polyphenol-based compounds are currently approved as standalone prescription medications for T2D, several polyphenol-rich extracts and supplements (such as resveratrol, green tea catechins, and curcumin) have been investigated as adjunctive therapies [95]. Meta-analyses of clinical trials indicate that these supplements, when added to standard care (i.e., metformin), can produce modest improvements in glycemic control, insulin sensitivity, and inflammatory markers [96]. They are generally well tolerated, with only mid gastrointestinal side effects reported at high doses [97]. However, consistent with the limitations noted above, current evidence is not yet sufficient to recommend routine clinical use, and long-term randomized controlled trials are needed. 

## 6. Impact of T2D on Dietary Polyphenol Absorption, Metabolism, and Transport

Building on the general properties and mechanisms discussed in Section 4 and the evidence of benefit in Section 5, this section explores the reciprocal side of the triangular interaction; namely, how T2D itself alters polyphenol absorption, metabolism, and bioavailability. It is important to note that the effects of T2D on polyphenol absorption, metabolism, and transport are not uniform across all compounds but vary depending on chemical class, molecular weight, and glycosylation status [89].

**Figure 1:**
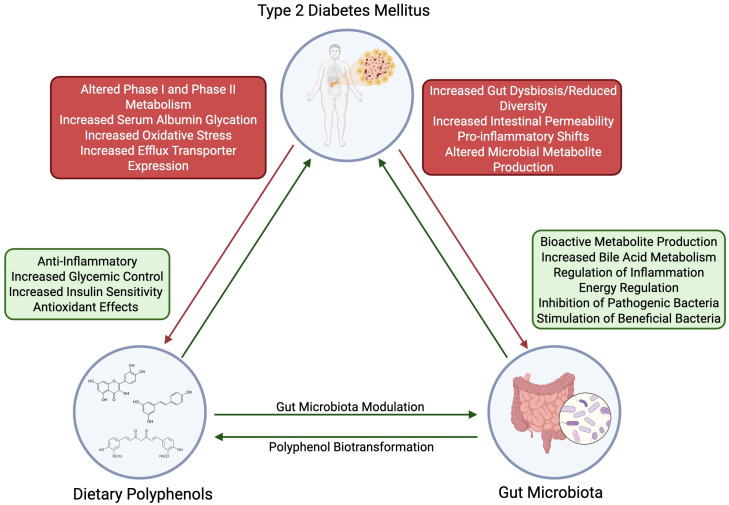
**Triangular interaction between dietary polyphenols, gut microbiota, and type 2 diabetes mellitus.** Dietary polyphenols exert potential therapeutic effects on the progression of T2D through anti-adipogenic, anti-inflammatory, and antioxidant mechanisms. Following ingestion, polyphenols undergo extensive biotransformation by the gut microbiota, yielding low-molecular-weight metabolites with enhanced bioavailability and bioactivity. In turn, these metabolites influence gut microbial composition by promoting the growth of beneficial taxa and suppressing pathogenic species, thereby establishing a reciprocal and dynamic relationship between dietary polyphenols and gut microbiota. Red arrows, along with the correspondingly colored annotations, indicate interactions or associations between components that may negatively affect host metabolic health. Green arrows and annotation boxes denote beneficial effects. This image was generated by using Biorender.com. Bayliss, E. (2026) https://BioRender.com/r9c9h17 (accessed on 11 March 2026).

While dietary polyphenols have attracted considerable interest for their potential role in the prevention and management of chronic disease, their therapeutic efficacy is heavily dependent on bioavailability, metabolism, and systemic transport to target tissues [95]. Because T2D can impair these pharmacokinetic processes, through mechanisms such as altered intestinal absorption, hepatic/renal metabolism, and transporter activity, it is essential to understand how the diabetic state modifies polyphenol handling (Figure 1) [98]. In a study using a Zucker diabetic fatty rat model, diabetes significantly altered the pharmacokinetics and bioavailability of grape seed polyphenols [99]. Plasma and brain polyphenol levels were lower in the diabetic rats, though the urinary loss of polyphenols was higher in this group. Some possible explanations for these differences may include delayed gastric emptying, intestinal dysmotility leading to malabsorption, gut microbial dysbiosis, and renal pathologies such as thickening of the basement membrane. Osmotic diuresis may further contribute to increased urinary polyphenol loss. Although microbial metabolites were not evaluated, these findings support the hypothesis that T2D influences the absorption, metabolism, and excretion of dietary polyphenols [99].

### 6.1. Effect on Absorption

T2D may alter the expression and activity of efflux transporters and enzymes involved in drug transport and metabolism. Hyperglycemia and chronically elevated levels of pro-inflammatory cytokines increase the expression of the efflux transporter P-glycoprotein (P-gp; multidrug resistance protein 1), which is expressed on the luminal surfaces of intestinal mucosa, liver canaliculi, renal tubules, and brain endothelial cells [100]. P-gp serves a broad protective function by pumping xenobiotics, drugs, and metabolites out of cells, but its upregulation in T2D may enhance the excretion and reduce intestinal uptake of many polyphenolic compounds [98]. Thus, the changes in transporter regulation under diabetic conditions may reduce systemic exposure to dietary polyphenols.

### 6.2. Effect on Metabolism

T2D can also impair hepatic and renal biotransformation of polyphenols. For example, the activity and expression of Phase II conjugating enzymes, such as uridine-5′-diphosphate-glucuronosyltransferase 2B7 (UGT2B7), are significantly reduced in individuals with diabetes [101]. A proposed mechanism involves systemic inflammation, which can suppress xenobiotic-metabolizing enzymes. In Sprague–Dawley rats, lipopolysaccharide-induced inflammation decreased hepatic sulfonation activity, illustrating a link between inflammatory signaling and impaired dietary compound metabolism [102]. These findings suggest that the chronic inflammatory milieu characteristic of T2D may compromise polyphenol metabolism and bioefficacy.

### 6.3. Effect on Transport

Alterations in protein binding and lipoprotein transport further affect polyphenol bioavailability in T2D. Plasma proteins from individuals with diabetes exhibit reduced capacity to bind polyphenols due to glycation-induced structural modifications [103]. This reduced binding increases the fraction of free polyphenols in circulation, rendering them more vulnerable to oxidation by ROS before reaching the target.

T2D also decreases the bioavailability of phenolic compounds transported by lipoproteins such as HDL, LDL, and VLDL [89]. Hyperglycemia-induced oxidative stress increases the susceptibility of these lipoprotein fractions to oxidative modification, impairing their ability to effectively transport polyphenols [89]. Altered protein- and lipid-mediated transport may therefore limit systemic distribution and diminish the functional effects of various dietary polyphenols in diabetes.

Understanding how T2D influences absorption, metabolism, and transport of polyphenols is crucial for optimizing their therapeutic potential [103]. Future research should integrate pharmacokinetics, gut microbiota profiling, and metabolic modeling to identify strategies that enhance polyphenol bioefficacy.

From a clinical perspective, interindividual variability in the absorption and metabolism of many dietary bioactive compounds presents a significant challenge when considering their therapeutic application. Unlike conventional medications, dietary compounds are consumed in non-standardized amounts and are influenced by differences in diet, comorbidities, medication use, and individual metabolic status [104]. These factors can significantly affect systemic exposure and therapeutic efficacy, complicating the translation of experimental findings into clinical recommendations. As a result, clinicians must exercise caution when interpreting claims regarding the metabolic benefits of nutraceuticals or dietary supplements.

These considerations emphasize the need for well-designed clinical trials when evaluating dietary interventions for metabolic disease. Randomized controlled studies that assess clinically meaningful outcomes, such as glycemic control, cardiovascular risk markers, and long-term disease progression, are necessary to determine whether experimental findings translate into measurable benefits for patients. Until such data are available, dietary bioactive compounds should be viewed primarily as supportive components of broader lifestyle interventions rather than substitutes for established medical therapies [105].

## 7. Interindividual Variation

While therapeutic modulation of the gut microbiota represents a promising strategy for the management of T2D, substantial interindividual variation remains a major challenge. Interindividual variation encompasses differences in genetic background, metabolic rate, gut microbiota composition, disease status, and environmental and lifestyle factors among individuals [106]. The bidirectional relationship between disease states, gut microbiome structure, and environmental exposures complicates efforts to predict host responses to dietary or pharmacologic interventions. In many cases, it remains unclear whether observed microbiome alterations are causal contributors to disease pathogenesis, consequences of the disease state, or reflections of confounding factors such as comorbid conditions, dietary habits, medication use, or antibiotic exposure [55].

Human dietary intervention studies consistently demonstrate marked interindividual variability in gut microbial composition and metabolic function. The gut microbiome is both highly dynamic and resilient; certain taxa may remain relatively stable, whereas others respond to environmental perturbations [56]. Consequently, interpretation of dietary intervention studies is often constrained by the lack of standardization, as many trials do not account for baseline gut microbiota composition or habitual dietary intake.

Interindividual variation in dietary polyphenol intake and the enzymatic capacity further contributes to wide variation in polyphenol bioactivity and bioefficacy [8]. This was demonstrated in a study assessing microbial bioconversion of red wine and black tea polyphenols, which revealed distinct metabolite and intermediate profiles among participants [107]. 

It is important to note that interindividual variability in the metabolism and bioefficacy of polyphenols is highly compound- and class-specific, with different polyphenols (i.e., resveratrol versus proanthocyanidins) showing markedly different responses depending on baseline microbiota composition and enzymatic capacity [75]. This suggests that each individual harbors a unique microbial metabolome that is shaped by strain-level differences and complex community interactions. Further, the identification of the specific microbial taxa responsible for particular metabolic conversions is complicated by cross-feeding interactions and redundant enzymatic pathways, whereby multiple species contribute to the production of similar metabolites [107].

Recognition of interindividual variation has driven the development of population-level and personalized approaches to microbiome research. Launched in 2007, the Human Microbiome Project (HMP) was among the first large-scale efforts to comprehensively integrate gene marker and metagenomic data across multiple body sites, including the gut, oral cavity, and vaginal microbiomes [108]. A key outcome of the HMP was the realization that taxonomic composition alone does not reliably predict host phenotype. Rather, microbial molecular function and strain-level diversity appear to provide more informative indicators of metabolic and health outcomes [109].

Expanding on this framework, longitudinal multi-omics studies have examined associations between gut microorganisms and host metabolic profiles in individuals with pre-diabetes. These analyses revealed correlations between gut microbiota composition and markers of insulin resistance, lipid metabolism, and systemic inflammation [110]. Insulin resistance, assessed using stead-state plasma glucose (SSPG), was positively associated with altered lipid metabolism, elevated systemic markers of inflammation, and increased abundance of the genus bacterial *Blautia*. However, the genera *Odoribacter*, *Oscillibacter*, and *Pseudoflavonifracter* were inversely associated with SSPG, suggesting a potential protective role. Microbial-derived metabolites, including indolelactic acid and hippuric acid, were inversely correlated with insulin resistance and served as biomarkers of increased microbiome diversity, which is generally associated with improved metabolic health and improved insulin sensitivity. Consistent with the inflammatory basis of T2D pathogenesis, associations between gut microbiota and cytokines were also observed with *Barnesiella* positively correlated with IL-1β, and *Faecalibacterium* inversely correlated with TNF-α [110].

Interindividual variability presents an important challenge to the management of metabolic diseases such as T2D. Patients with similar clinical characteristics often demonstrate markedly different responses to dietary interventions, pharmacologic therapies, and lifestyle modifications, complicating the development of broadly effective treatment strategies. This variability has contributed to growing interest in precision medicine approaches that aim to tailor prevention and treatment strategies to individual patient profiles. Advances in multi-omics technologies and large-scale clinical datasets may eventually enable clinicians to better stratify patients and identify those most likely to benefit from specific therapeutic or dietary interventions. Although these approaches remain largely investigational, integrating personalized strategies into diabetes care holds promise for long-term disease management and therapeutic outcomes [111].

## 8. Conclusions

When the gut microbiome is in homeostasis, it supports nutrient metabolism, immune function, and intestinal barrier integrity while generating microbial metabolites that function as signaling molecules and potential biomarkers of health and disease. Conversely, gut dysbiosis, characterized by reduced microbial diversity and altered taxonomic composition, disrupts this balance and contributes to a cycle of inflammation and increased intestinal permeability. At the same time, it is worth noting that identifying or characterizing dysbiosis is a challenge due to the extent of the variability that exists in otherwise “normal” communities. This allows gut-derived toxins and antigens to enter systemic circulation, perpetuating chronic, low-grade inflammation that promotes insulin resistance and metabolic dysfunction.

Disease states such as T2D can further influence the absorption, metabolism, and transport of dietary polyphenols. Chronic hyperglycemia and systemic inflammation alter Phase I and Phase II enzyme activity, reduce polyphenol bioavailability, and induce the upregulation of efflux transporters such as P-gp, thereby decreasing intestinal absorption. Additionally, glycation of plasma proteins reduces their binding affinity for polyphenols, diminishing antioxidant delivery to target tissues and potentially attenuating biological efficacy.

These findings collectively highlight the gut microbiota as a central node of interaction between dietary polyphenols and metabolic health. The close interdependence between metabolic disease and gut dysbiosis suggests shared etiological influences, which include dietary patterns, genetic predisposition, and other environmental factors. Targeted modulation of the gut microbiome by using polyphenol-based interventions therefore represents a promising avenue for the prevention and management of T2D. However, factors such as polyphenol concentration, bioavailability, dosing, and interindividual variability in microbiome composition, diet, and comorbid conditions must be carefully considered.

In conclusion, the findings presented in this review support our hypothesis of a dynamic bidirectional triangular interaction among dietary polyphenols, the gut microbiota, and T2D. Dietary polyphenols beneficially modulate gut microbial composition and function, leading to increased production of SCFAs, reduced systemic inflammation, strengthened intestinal barrier integrity, and improved insulin sensitivity. Conversely, the diabetic state and associated gut dysbiosis impair polyphenol absorption, metabolism, and bioavailability, potentially limiting their therapeutic efficacy. This reciprocal relationship highlights the promise of polyphenol-rich dietary interventions as complementary strategies for the prevention and management of T2D. Future well-designed clinical trials that account for interindividual variability in microbiome composition will be essential to translate these insights into effective personalized nutrition and therapeutic approaches. 

## Figures and Tables

**Table 1:** Major classes of dietary polyphenols: chemical structures, food sources, microbial metabolism, bioavailability in Type 2 diabetes (T2D), and mechanisms of action. Chemical structures were sourced from the Merck Index database (Merck & Co., Rahway, NJ, USA).

Phenol Class	Representative Compounds	Chemical Structure/Key Features	Primary Food Sources	Key Microbial Taxa Involved in Biotransformation	Bioavailability and T2D-Specific Barriers	Effects on Gut Microbiota	Anti-Diabetic Mechanisms and T2D’s Outcomes
Flavonoids	Quercetin, Catechins (EGCG), Anthocyanins	Quercetin 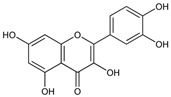	Onions, apples, berries, tea, cocoa, grapes	*Bifidobacterium*, *Lactobacillus*, *Lachnospiraceae*, *Akkermansia*	Moderate absorption; deglycosylation required. T2D reduces bioavailability via increased P-gp efflux and altered Phase II conjugation	Increase beneficial taxa (*Akkermansa*, *Bifidobacterium*); increase SCFA production; decrease pathogenic bacteria	Activate AMPK, improve insulin sensitivity, reduce inflammation (decrease NF-kB, TNF-α), antioxidant effects
Phenolic Acids	Chlorogenic acid, Gallic acid, Caffeic acid	Chlorogenic acid 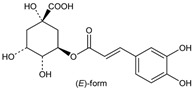	Green coffee beans, blueberries, sunflower seeds, tea, zucchini	*Bifidobacterium*, *Lactobacillus*, *Bacteroides*	Moderate to high absorption due to low molecular weight. Gut dysbiosis reduces biotransformtion; increased P-gp efflux and accelerated Phase II conjugation reduce bioavailability	Increase beneficial taxa (*Akkermansa*, *Bifidobacterium*, *Lactobacillus*); increase SCFA production; suppress pro-inflammatory taxa	Activate AMPK, improve insulin sensitivity, reduce inflammation (decrease NF-kB, TNF-α), antioxidant effects, improved glycemic control
Stilbenes	Resveratrol	Resveratrol 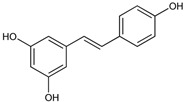	Grapes, red wine, blueberries, peanuts, cocoa	*Bifidobacterium*, *Lactobacillus*, *Bacteroides*, *Slackia*	Poor parent bioavailability leading to rapid Phase II conjugation and increased P-gp efflux. Biotransformation limited by dysbiosis	Moderate restoration of microbial diversity, increase SCFA production, reduces pro-inflammatory taxa	Activate AMPK, improve insulin sensitivity, effectively reduce inflammation (decrease NF-kB, TNF-α), antioxidant effects, improved glycemic control
Lignans	Secoisolariciresinol, Matairesinol, Enterodiol, Enterolactone	Enterolactone 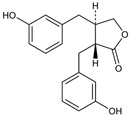	Flaxseed, sesame seeds, whole grains, legumes	*Eggerthella*, *Bifidobacterium*, *Lactobacillus, Clostridium*	Poor absorption; dysbiosis decreasing converting bacteria. Possible Phase II conjugation acceleration.	Increases beneficial taxa (*Bifidobacterium*, *Lactobacillus*), increases SCFA production, reduces pro-inflammatory taxa, strengthens gut barrier integrity.	Activate AMPK, improve insulin sensitivity, reduce inflammation (decrease NF-kB, TNF-α), modulates glucose and lipid metabolism as a phytoestrogen, improved glycemic control
Tannins	Gallic acid, Ellagic acid, Tannic acid, Procyanidin B2, Epicatechin	Ellagic acid 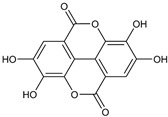	Apples, legumes, pomegranates, rasberries, grapes, cocoa, tea	*Bifidobacterium*, *Lactobacillus*, *Eggerthella, Gordonibacter*, *Clostridium*	Poor intestinal absorption due to large molecular weight. Dysbiosis decreases bacteria for biotransformation. May modulate P-gp efflux transport to promote absorption. Possible acceleration of Phase II conjugation	Increases beneficial taxa (*Bifidobacterium*, *Lactobacillus*), increases SCFA production, reduces pro-inflammatory taxa, strengthens gut barrier integrity	Increase butyrate production to improve insulin sensitivity, reduce inflammation (decrease NF-kB, TNF-α), antioxidant effects, modulate glucose metabolism, improve glycemic control

## Data Availability

No new data were created or analyzed in this study. Data sharing is not applicable to this article.

## Abbreviations

The following abbreviations are used in this manuscript:

T2D	Type 2 Diabetes Mellitus
OH	Hydroxyl
ROS	Reactive Oxygen Species
GM	Gut Microbiota
BCAA	Branched-Chain Amino Acid
FMT	Fecal Microbiome Transplantation
JNK	Jun N-Terminal Kinases
IL-1	Interleukin-1
IL-6	Interleukin-6
TNF- α	Tumor Necrosis Factor-Alpha
FPR	Formyl Peptide Receptor
O_2_^−^	Superoxide
H_2_O_2_	Peroxide
SCFA	Short-Chain Fatty Acid
AMPK	AMP-Activated Protein Kinase
Nlrp3	Pyrin Domain-Containing Protein 3
PKM2	Pyruvate Kinase M2
IL-22	Interleukin-22
TLR4	Toll-Like Receptor 4
LBP	Lipopolysaccharide Binding Protein
IL-18	Interleukin-18
P-gp	P-Glycoprotein
UGT2B7	Uridine-5′-Diphosphate-GlucuronosylTransferase 2B7
HMP	Human Microbiome Project
SSPG	Steady-State Plasma Glucose
